# A Fast and Specific Alignment Method for Minisatellite Maps

**Published:** 2007-02-22

**Authors:** Sèverine Bérard, François Nicolas, Jérôme Buard, Olivier Gascuel, Eric Rivals

**Affiliations:** 1 INRA, Département MIA, Toulouse, France; 2 LIRMM, UMR 5506 CNRS-Université de Montpellier II, Montpellier, France; 3 Institut de Génétique Humaine, UPR-CNRS 1142, Montpellier, France

**Keywords:** VNTR, tandem repeat, tandem duplication, variable costs, dynamic programming, sequence comparison

## Abstract

**Background:**

Variable minisatellites count among the most polymorphic markers of eukaryotic and prokaryotic genomes. This variability can affect gene coding regions, like in the prion protein gene, or gene regulation regions, like for the cystatin B gene, and be associated or implicated in diseases: the Creutzfeld-Jakob disease and the myoclonus epilepsy type 1, for our examples. When it affects neutrally evolving regions, the polymorphism in length (*i.e.,* in number of copies) of minisatellites proved useful in population genetics.

**Motivation:**

In these tandem repeat sequences, different mutational mechanisms let the number of copies, as well as the copies themselves, vary. Especially, the interspersion of events of tandem duplication/contraction and of punctual mutation makes the succession of variant repeats much more informative than the sole allele length. To exploit this information requires the ability to align minisatellite alleles by accounting for both punctual mutations and tandem duplications.

**Results:**

We propose a minisatellite maps alignment program that improves on previous solutions. Our new program is faster, simpler, considers an extended evolutionary model, and is available to the community. We test it on the data set of 609 alleles of the MSY1 (DYF155S1) human minisatellite and confirm its ability to recover known evolutionary signals. Our experiments highlight that the informativeness of minisatellites resides in their length and composition polymorphisms. Exploiting both simultaneously is critical to unravel the implications of variable minisatellites in the control of gene expression and diseases.

## 1. Introduction

### 1.1. Polymorphic tandem repeats

Polymorphic tandem repeat loci, also known as Variable Number Tandem Repeats (VNTR), are widely used as genetic markers in population genetics, gene mapping, and forensic medicine (Jeffreys et al. 1985). Microsatellites, which are tandem repeats of a 1–6 bp long motif, show a frequent variability in their number of repeats. The expansion in some triplet microsatellites forms the molecular basis of a dozen inherited neurodegenerative diseases (Cummings and Zoghbi, 2000). Polymorphism was observed in another class of tandem repeats with motif size in the 7–100 bp range, the minisatellites. Unlike microsatellites, unstable minisatellites display not only repeat number variability, but also sequence heterogeneity between repeats. The succession of the variant repeats along a minisatellite allele can be obtained by a specific method called Minisatellite Variant Repeat-PCR (Jeffreys et al. 1991) (MVR-PCR). It provides a *MVR map*: a string of symbols in which each symbol represents a different variant of the minisatellite repeat unit. The correspondence between the minisatellite sequence and the map is illustrated below.

**Example 1:** We illustrate the correspondence between the DNA sequence and the MVR map with a fictitious DNA sequence, in which for pagination reason, the repeats are only 7 pb long.

Sequence: CGGCGAT CGGCGAC CGGAGAT CGGCGAT CGGCGAT CGGAGAT CGACGAT

Alphabet of variants: a = CGGCGAT b = CGGCGAC c = CGGAGAT d = CGACGAT

Corresponding MVR map: a b c a a c d

Variable minisatellites were also shown to be involved in the development of inherited diseases: either by influencing gene transcription, like in the progressive myoclonus epilepsy type 1, or by being part of a coding sequence, like in the prion protein gene, which is responsible for the Creutzfeld-Jakob disease (Buard and Jeffreys, 1997; Bois and Jeffreys, 1999). The informativeness of unstable minisatellites has led to their widespread use for individual identification, parentage analysis (Jeffreys et al. 1985), and for discrimination between bacterial strains, including anthrax strains (Le Flèche et al. 2001).

### 1.2. Minisatellite variability and turnover processes

Comparison between the internal structure of alleles has been shown to be the key to elucidate the mechanism of minisatellite expansion and deletion for several human autosomal GC-rich minisatellite loci. Complex rearrangements involving transfer of groups of repeats between alleles as well as intra-allelic duplications have been deduced by alignment “by eye” between MVR maps of progenitor and new length alleles (Buard and Vergnaud, 1994; Jeffreys et al. 1994; May et al. 1996; Stead and Jeffreys, 2000; Tamaki et al. 1999). However, as the rearrangements may completely reshuffle the repeat array in a single generation (Tamaki et al. 1999), the parental relationships is easily lost when studying more distantly related alleles (*e.g.,* from different populations). This renders unstable minisatellites showing a significant fraction of mutations as highly complex rearrangements involving inter-allelic exchanges inadequate tools for population-wide evolutionary studies. Among those minisatellites are hypervariable GC-rich autosomal loci in human.

Many variable minisatellites with mutation rate below 1% per gamete have been reported in human and in other species. For instance, in the human insulin minisatellite, the variation is mainly due to the gain or loss of one repeat, which occurs at a rate of 10^−3^ per gamete, while complex inter-allelic rearrangements happen at much lower frequency (10^−5^) (Stead and Jeffreys, 2000). In other eukaryotic species, no hypervariable minisatellites were discovered (Bois and Jeffreys, 1999). Detailed investigation of murine minisatellites provides evidence of a variability dominated again by simple intra-allelic duplication occuring at a rate of 10^−4^ per gamete (Bois et al. 1998). This suggests that these minisatellites can serve for evolutionary population studies. Indeed, alignments between MVR maps have also been used to deduce the evolutionary relationships between alleles for the study of recent human population history (Alonso and Armour, 1998; Armour et al. 1993; Hurles et al. 1998; Jobling et al. 1998; Stead and Jeffreys, 2002). Both the potential investigative power of variable minisatellites for evolutionary studies and their use for identification has been limited by the lack of computerized methods to objectively compare alleles.

The evolutionary events at work in minisatellite turnover are divided into inter- and intra-allelic events. Inter-allelic events mean rearrangements between the two alleles of an autosomal minisatellite, while intra-allelic events comprise the amplification and contraction of a repeat or of a block of consecutive repeats, as well as the nucleotidic mutations inside the repeats. For the acquisition of MVR maps, the limits of the variant repeats are chosen arbitrarily, and when comparing maps, duplication events are assumed to copy complete variants (and not, for example, a variant and the half of the following variant). However, the mechanisms of DNA duplication may duplicate any segment of DNA inside the minisatellite, and their templates do not always correspond to complete repeats. Therefore, comparison of minisatellite maps relies on the assumption that the boundaries of the variant repeats are fixed and that duplications copy complete variants. This assumption (discussed from the algorithmic view-point in (Benson and Dong, 1999) and (Rivals, 2004)) may not be satisfied for all minisatellites, but seems generally valid for polymorphic tandem repeat loci.

### 1.3. Existing algorithms for minisatellite comparison

Several methods to compare MVR maps were published recently. All of them consider solely intra-allelic evolution. The statistical similarity measure defined in (Brion et al. 2002) computes a weighted sum of the number of shared variants when the two maps are compared at different relative positions. This measure depends to a great extent on the weight function used; in addition, the distance based on it is not a metric, which is a serious drawback for phylogenetic reconstruction. Alignment of minisatellite maps under a specific evolutionary model that considers indels, substitutions, but also tandem duplications and contractions of variants was first described in (Bérard and Rivals, 2003). There, as well as in (Behzadi and Steyaert, 2004; Behzadi and Steyaert, 2005), duplications and contractions are limited to a single variant (*e.g., abc* → *abbc*); in other words, the duplication of a block of consecutive variants (*e.g., abcd* → *abcbcd*) is not allowed as a single event. Compared to classical sequence alignment, the result of a series of events on a map is order dependent (*e.g.,* duplication + substitution ≠ substitution + duplication), which makes the computation more complex. In (Bérard and Rivals, 2003), the proposed alignment procedure accounts for these dependencies and computes an optimal alignment under a model where all mutations have the same cost.

Other works aim at improving the efficiency of this algorithm. A first method performs a Run-Length Encoding of the maps to reduce the complexity of the procedure (Behzadi and Steyaert, 2004). Run-Length Encoding (RLE) is a data compression technique in which a stretch of the same letter is coded as a power of this letter, *e.g., a*^3^ instead of *aaa*. The algorithm of (Behzadi and Steyaert, 2005), performs the computation of the alignment distance in cubic time using a model that allows for variable mutation costs, *i.e.,* costs that depend on the variants involved. However, unlike in (Bérard and Rivals, 2003), even if the elementary costs are symmetrical, the distance computed is not symmetrical, which prevents it from being a metric. Recently, an algorithm that accounts for duplication and excision of blocks in the alignment of DNA sequences has been published (Sammeth et al. 2005). Here, a more complex evolutionary model is considered as a block of several variants may be duplicated in a single event; this explains why the algorithm complexity is exponential. In addition, none of these methods, (Behzadi and Steyaert, 2004), (Behzadi and Steyaert, 2005), (Sammeth et al. 2005), is available to the biological community. Thus, we designed and implemented an exact algorithm to align maps that uses RLE for efficiency and computes an alignment distance that is a metric. Our mutational model authorizes variable mutation costs as in (Behzadi and Steyaert, 2005).

### 1.4. Biological validation

To validate our algorithm, we choose a data set from a minisatellite on the human paternally inherited Y chromosome, called MSY1. Most of the Y chromosome is haploid and thus escapes recombination. It contains the whole range of polymorphic systems observed in the human genome with mutation rates varying from 5.10^−7^% for base substitutions to 3.8% for the hypervariable minisatellite MSY1 (DYF155S1) (Andreassen et al. 2002). Probably due to its obligatory intra-allelic mode of mutation (no complex rearrangement between alleles) and to its AT-richness, MSY1 evolution is dominated by the gain or loss of a single variant (Andreassen et al. 2002), and is thus adequate to the model we hypothesized in this work. As a result, MSY1 alleles have a simple modular structure of variant repeats compared with the intermingled pattern of variants resulting from recombinational exchanges at hypervariable autosomal minisatellites (Figure 1 and also (Jobling et al. 1998)). Homogenization of the variants along the array has been observed at the locus MSY1, suggesting the occurrence of complex rearrangements like at autosomal loci. However, this phenomenon is restricted to alleles belonging to a single Y haplogroup (Bouzekri et al. 1998).

MSY1 haploid nature also avoids physical separation of the two alleles, which is a technical bottleneck for autosomal minisatellites. Consequently, a large number of MSY1 alleles originating from males all over the world were typed by several authors. The Y chromosome represents a unique system for comparing inter-chromosome relationships established with MVR maps and those deduced from more stable markers. (Jobling and Tyler-Smith, 2000), studied the evolutionary relationships between stable markers on the Y chromosome. They defined haplogroups using these markers and reconstructed a most parsimonious tree for them. The availability of known precise relationships between a large set of alleles is a major reason for the choice of MSY1.

The purpose of our experiments was to investigate whether known phylogenetic relationships between Y chromosomes could be independently recovered from the alignments of MSY1 MVR maps. Moreover, as the MSY1 MVR maps were obtained from individuals taken from different populations, we could check whether the coalescence of MSY1 haplotypes we inferred inside a Y chromosomal haplogroup agrees with the populations’ relationships. We analyzed 609 alleles of MSY1 with our program and found inter- and intra-haplogroup relationships that are consistent with the evolution of the Y chromosome.

### 1.5. Article overview

The remaining of this paper is organized as follows. In Section 2, we provide a notation and define an evolutionary model for minisatellites. Section 3 explains the alignment algorithm. Section 4 describes the experiments performed on 609 alleles of the MSY1 human haploid minisatellite to validate our program MS_Align. We conclude in Section 5.

## 2. Evolutionary Model

### 2.1. Notations

Let ∑ be a finite alphabet of variants. A map *s* is a string of |*s*| characters of ∑ indexed from 1 to |*s*|. The empty string is denoted by ɛ. For any integers *i*, *j*, 1 ≤ *i* ≤ *j* ≤ |*s*|, *s*[*i*] denotes the *i*^th^ symbol of *s*, and *s*[*i..j*]:= *s*[*i*]…*s*[*j*] the *substring* between positions *i* and *j* of *s*. Throughout the article, let *r*, *s* be two maps over ∑ of length *m* and *n*, respectively.

### 2.2. Evolutionary model

Tandem repeat sequences undergo two kinds of evolutionary events: point mutations (substitutions and indels) that act on nucleotides and modify the minisatellites variants, and specific events acting on a complete variant: tandem *amplification* and its opposite, tandem *contraction*. The change of a variant into another, be it caused by a nucleotidic substitution or by an indel, is called a *mutation* in the sequel. An amplification duplicates a variant and put the generated copy at its side. Our evolutionary model also takes into account the events of insertion and deletion of one variant. Therefore, it contains five evolutionary events on variants: mutation of a variant into another, insertion, deletion, amplification, and contraction. We take into account amplifications and contractions of one variant, *i.e.,* producing/deleting only one copy at a time; these are the most frequent events at MSY1 (Andreassen et al. 2002).

**Example 2:** Illustration of the operations, where a, b, c, d are variants:

Insertion of d at position 3: abc → abdc;

Amplification of b: abc → abbc;

Deletion of b: abc → ac;

Contraction of b: abbc → abc;

Mutation of b into d: abc → adc.

These five operations are gathered under the term *elementary operations* as they involve one variant at a time. A positive cost is associated to each operation. A succession of events that transforms *s* into *r* is called an alignment between *s* and *r*. The alignment cost is the sum of the costs of the operations it contains. We denote each cost by the uppercase initial of the corresponding operation: *I* for insertion, *D* for deletion, *A* for amplification and *C* for contraction. The mutation cost depends on the variants. We denote ℳ(*a*, *b*) the mutation cost of variant *a* into variant *b*; ℳ(*a*, *b*): = 0 if and only if *a* = *b*. The costs abide by the triangular inequality: ∀*a*,*b*,*c* ∈ ∑ ∪ {ɛ}, ℳ(*a*, *b*) ≤ ℳ(*a*, *c*) + ℳ(*c*, *b*), where ℳ(*a*, ɛ): = *D* and ℳ(ɛ, *a*): = *I*. Here, we consider a symmetric model where opposite operations have the same cost: *I* = *D*, *A* = *C* and ∀*a*, *b* ∈ ∑, ℳ(*a*, *b*) = ℳ(*b*, *a*). As the frequency of amplifications and contractions on minisatellite maps is higher than the frequency of other operations, these two events have a lower cost: *A*, *C* ≪*D*, *I*, ℳ(*a*, *b*),∀*a*, *b* ∈ ∑.

The alignment cost under this model is a metric (see the proof of (Bérard and Rivals, 2003)). It matters when using the cost as a distance, for example in phylogenetic reconstruction.

#### 2.2.1 Segmental operations

The set of operations of our evolutionary model induces “long distance” dependencies. As an example, let us consider the following two generations of sequence *aba* from character *a*:

1. $a\overset{A}{\to }a\;a\overset{A}{\to }a\;a\;a\overset{ℳ(a\;b)}{\to }a\;b\;a$
2. $\begin{array}{l} a\overset{A}{\to }a\;a\overset{ℳ(a\;b)}{\to }a\;b\overset{A}{\to }a\;b\;b\\ \overset{ℳ(b\;a)}{\to }a\;b\;a. \end{array}$

The first one contains 2 amplifications and 1 mutation though the second one contains 2 amplifications and 2 mutations. In the first generation, the last character *a* of *aba* appeared before the *b* and was produced by an amplification of a variant *a*, which is no longer at its side. This example illustrates the non-commutativity of operations, *i.e.,* that the order in which the operations are applied matters. A procedure that would consider the variants independently from left to right, as the second generation scheme, cannot always find the optimal alignment. To handle such cases, we define two supplementary operations: *Generation of a substring* and *Compression of a substring* These operations enable to align straightly one variant with a whole substring, taking into account the optimal application order of elementary operations. Generation and compression of a substring are symmetrical and gathered under the term *segmental operations*.

**Example 3:** An illustration of segmental operations with the alphabet of variants being a, b, c, d is shown on Figure 4. From the rightmost occurrence of b in the upper map, we generate the substring bbcaccbb in the lower map. In a generation, any symbol of the generated substring (in the lower map) is an offspring of the source symbol (in the upper map). The sequence of operations is represented as a tree whose root is the b in the upper map, and whose leaves ordered from left to right are the characters of the generated substring. If we consider the tree bottom-up, then it represents the compression of the substring bbcaccbb into the symbol b.

## 3. Algorithm

Our alignment algorithm is composed of two phases:

1. Computation of the costs of all possible segmental operations in each map;
2. Alignment of the 2 maps by dynamic programming, taking into account both the elementary and the segmental operations computed at step 1.

### 3.1. First phase

This phase precomputes the costs of all segmental operations for each map. For a map, say *s*, it stores these costs in a three dimensional table 𝒞*_s_* indexed on (∑ ∪ {ɛ}) × [1..*n*] × [1..*n*] and defined as follows: ∀*i* ≤ *j* ∈[1..*n*] and ∀*a* ∈ ∑, 𝒞*_s_* (*a*, *i*, *j*) is the minimum cost required to generate *s*[*i..j*] from *a*, and 𝒞*_s_*(ɛ, *i*, *j*) is the minimum cost to generate *s*[*i..j*] from the empty string. As generation and compression are symmetrical, 𝒞*_s_*(*a*, *i*, *j*), resp. 𝒞*_s_*(ɛ, *i*, *j*), is also the optimal cost to compress *s*[*i..j*] in *a*, resp. in ɛ. Only half of the matrix is filled, since we compute the entries (·, *i*, *j*) such that *i* ≤ *j*. We utilize an intermediate table, 𝒮*_s_*, indexed on (∑ ∪ {ɛ}) × [1..*n*] × [1..*n*], in which each entry 𝒮*_s_*(*a*, *i*, *j*) is the minimum cost required to generate *s*[*i..j*] from *a* without mutation as first operation. The rationale is the following property: there exists an optimal sequence of operations that generates *s*[*i..j*] from the symbol *a,* which does not start by two successive mutations. Indeed, if it starts by changing *a* into *b*, and then changing *b* into *c*, then mutating directly *a* into *c* would not cost more. Using the two tables 𝒮*_s_* and 𝒞*_s_* allows to avoid computing such non-optimal generations, and improves the time complexity.

In 𝒮*_s_*, only the cells located strictly above the main diagonal are used (*i.e.,* such that *i* < *j*). The recurrences to fill jointly tables 𝒞*_s_* and 𝒮*_s_* are:

$$\begin{array}{l} \mathrm{Initialization}:C_{s}(ɛ,i,i):=I;\forall a\in \Sigma \;C_{s}(a,i,i):=ℳ(a,s[i]\\ \mathrm{Recurrence}:\forall i<j\{\begin{array}{l} (a)\;\Sigma_{s}(a,i,j):=\text{min}\{\begin{array}{ll} A+\overset{j-1}{\underset{k=i}{\text{min}}}\;({\text{X}}_{s}(a,i,k)+{\text{X}}_{s}(a,k+1,j)) & \left(a_{1}\right)\\ \overset{j-1}{\underset{k=i}{\text{min}}}\;({\text{X}}_{s}(a,i,k)+{\text{X}}_{s}(ɛ,k+1,j)) & \left(a_{2}\right)\\ \overset{j-1}{\underset{k=i}{\text{min}}}\;({\text{X}}_{s}(ɛ,i,k)+{\text{X}}_{s}(a,k+1,j)) & \left(a_{3}\right) \end{array}\\ \left(b)\;{\text{X}}_{s}(a,i,j):=\underset{b\;\in \;\Sigma }{\text{min}}(\text{M}(a,b)+\Sigma_{s}(b,i,j)\right)\\ \left(c)\;{\text{X}}_{s}(ɛ,i,j):=\underset{b\;\in \;\Sigma }{\text{min}}(I+\Sigma_{s}(b,i,j,)\right) \end{array} \end{array}$$

For each pair (*i*, *j*), corresponding to the substring *s*[*i..j*], and each letter *a*, we first compute 𝒮*_s_* (*a*, *i*, *j*) by Equation (*a*), and then 𝒞*_s_* (*a*, *i*, *j*) by equations (*b*) and (*c*).

The initialization computes the generation costs of all one-character substrings, *i.e.,* for all pairs (*i*, *j*) such that *i* = *j*. In this case, the segmental operation corresponds to an elementary operation: either an insertion if we start from ɛ (𝒞*_s_* (ɛ, *i*, *i*): = *I*), or a mutation if we start from a symbol (𝒞*_s_* (*a*, *i*, *i*): = ℳ(*a*, *s*[*i*])). The initialization fills the cells of the main diagonal, 𝒞*_s_* (·, *i*, *i*).

The main recurrence is decomposed in 3 cases, it uses the intermediate table 𝒮*_s_*, and fills 𝒞*_s_* and 𝒮*_s_* diagonal by diagonal.

- For the table 𝒮*_s_*, the generation shall not start with a mutation, for the reason explained above. The substring *s*[*i..j*] is decomposed in two non empty parts: a prefix, *s*[*i..k*], and a suffix, *s*[*k* + 1..*j*], and all possible values of *k* in [*i.. j* − 1] are considered. As *j* > *i*, each of them is at least one character long. The computation of 𝒮*_s_*(*a*, *i*, *j*) is obtained by taking the minimum among the three possibilities to generate *s*[*i..j*] from *a*:
  (a1) Duplicate the symbol *a*, which yields *aa*, and then generate the complementary prefix and suffix from each *a*, which is accounted for by summing 𝒞*_s_* (*a*, *i*, *k*) and 𝒞*_s_* (*a*, *k* + 1, *j*).
  (a2) Generate the prefix *s*[*i..k*] from symbol *a*, and generate the suffix *s*[*k* + 1..*j*] from ɛ,
  (a3) or symmetrically, generate the prefix from ɛ, and generate the suffix from symbol *a*.
- To generate *s*[*i..j*] from symbol *a*, we consider the sequences of operations starting with a mutation of *a* into *b*, over all possible symbol *b*, (eventually at null cost when *b* = *a*), and followed by the generation *s*[*i..j*] from this symbol *b*. For the latter cost, we use the intermediate table 𝒮*_s_*.
- Finally, to generate *s*[*i..j*] from the empty string ɛ, one has to insert a first character, say *b*, and then generate *s*[*i..j*] from *b*. By a similar property as the one detailed above, an optimal generation of *s*[*i..j*] from the inserted symbol *b* cannot begin with a mutation of *b* into any other symbol *c* (otherwise, one could have straightly inserted the desired symbol). Thus, the generation cost of *s*[*i..j*] from *b* is looked up in 𝒮*_s_*.

This preprocessing is performed on both *s* and *r* to obtain the tables 𝒞*_s_* and 𝒞*_r_* in time *O*(*p*^3^|∑|), with *p* = max(*n*, *m*). Yet, one can speed up the preprocessing by exploiting the repetitiveness of the sequences, and by using instead of sequence *s*, its *Run-Length Encoded* (RLE) version denoted *s̃. E.g*., if *s* = *aaaabbcccaaa*, then *s̃* = *a*^4^*b*^2^*c*^3^*a*^3^ and, |*s̃*| = 4 (though |*s*| = 12). The precomputing of *C_s̃_* is illustrated in Figure 2(top) and takes *O*(|*s̃*|^3^|∑|). Moreover, one can deduce any entry of 𝒞*_s_* from *C_s̃_* in constant time with a precomputed table *σ*, which is a by-product of the computation of the RLE of *s*. For any position *i* in *s*, *σ* (*i*) gives the number of the block of identical symbols in *s* to which *s*[*i*] belongs to (Figure 2 bottom-right). Indeed, generating *s*[*i..j*] from a symbol *a* is equivalent to first generating *s̃*[*σ*(*i*)..*σ*(*j*)] from *a*, and then amplifying the variants inside the blocks. Thus, 𝒞*_s_* (*a*, *i*, *j*) equals *C_s̃_*(*a*, *σ*(*i*), *σ*(*j*)), plus the amplification cost times the length of *s*[*i..j*] minus the number of blocks in *s*[*i..j*], which is the formula given in Figure 2 (bottom-left). In the MSY1 dataset, as |*s̃*| ≪ *n*, this trick speeds up by 6 the global computing time (phases 1 and 2), an improvement of about 40% over the time of MS_Align Version 1 (Bérard and Rivals, 2003).

### 3.2. Second phase

To align globally maps *s* and *r*, we construct a dynamic programming matrix 𝒜, indexed on [0..*n*] × [0..*m*]. 𝒜(*i*, *j*) is the alignment distance between the prefixes *s*[1..*i*] and *r*[1..*j*]; the distance between the two maps is 𝒜(*n*, *m*). The difference with classical alignment comes from segmental operations, which force us to consider long range dependencies in 𝒜. An optimal alignment of maps *s*[1..*i*] and *r*[1..*j*] (*i* >1 and *j* > 1) is decomposed in an optimal alignment of smaller prefixes and one final operation, for which two symmetrical cases arise:

1. the compression of a suffix of *s*, *s*[*l..i*], either into its first character *s*[*l*], which is aligned with *r*[*j*] (Figure 3(*a*)), or into character *r*[*j*] of *r* (Figure 3(*b*)),
2. the generation of a suffix of *r*, *r*[*l*′..*j*], from character *r*[*l*′] of *r*, or from character *s*[*i*] of *s*.

This explains the following recurrence for 𝒜 which takes the minimum between 4 possibilities:

$$\begin{array}{l} \mathrm{Initialization}:A(0,0):=0;A(1,0):=D;A(0,1):=I\\ \mathrm{Recurrence}:A(i,j):=\text{min}\{\begin{array}{ll} A(l,j)+C_{s}(s[l],l,i) & \text{if }i>1,\forall l\in [1..i-1]\\ A(l-1,j-1)+C_{s}(r[j],l,i) & \text{if }i>0\;\text{and}\;j>0,\forall l\in [1..i]\\ A(i,l^{\prime })+C_{r}(r[l^{\prime }],l^{\prime },j) & \text{if }j>1,\forall l^{\prime }\in [1..j-1]\\ A(i-1,l^{\prime }-1)+C_{r}(s[i],l^{\prime },j) & \text{if }j>0\;\text{and }i>0,\forall l^{\prime }\in [1..j] \end{array} \end{array}$$

Remember that elementary operations are trivial cases of segmental operations as illustrated in Figure 3: when *l* = *i* − 1 for deletion and contraction in case (*a*), when *l* = *i* for mutation in case (*b*). The time complexity is *O*(*p*^3^) with *p* = max(*n*, *m*) for this phase, and *O*(*p̃*^3^|∑| + *p*^3^) for the complete algorithm, where *p̃* = max(|*s̃*|, |*r̃*|).

### 3.3. Improvements over the algorithm of (Bérard and Rivals, 2003)

We provide a novel program (MS_Align Version 2) to compare minisatellite maps. The set of events authorized comprises all elementary events that occur in the intra-allelic evolution of a minisatellite. The evolutionary model has been extended to account for variable mutation costs. Note that the model can be extended to enable all costs to be variant dependent. In the original model ℳ(*a*, *b*) was identical regardless of *a*, *b* ∈ ∑ such that *a* ≠ *b*. Second, we have generalized “arches” operations to generation and compression of all substrings, which yields a simpler formulation of the algorithm. Finally, by using RLE maps we reduced the running time, although the worst-case complexity is higher because of the model. Thus, the algorithm is now faster and so more exploitable. Figure 4 displays an alignment resulting from our algorithm. This figure shows at the same time the alignment and the scenarios associated to the segmental operations.

### 3.4. Further direction for algorithmical refinements

We also envisaged reducing the complexity of our algorithm *O*(*p̃*^3^|∑| + *p*^3^) to *O*(*p̃*^3^|∑|). This time complexity could be achieved if the 2nd phase was done on RLE sequences. This appears to be difficult. Indeed, it would only be possible if the blocks do not overlap in an optimal alignment between two maps. However, in the counter-example shown below, where *s*:= *a*^990^*b*^1^*a*^10^ and *r*:= *a*^10^*b*^1^*a*^990^, the unique optimal alignment^1^ costs 2 × ℳ(*a*, *b*), and, it contains two large *a* blocks overlapping each other.


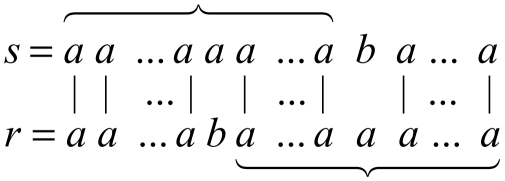


Nevertheless, in practice MS_Align can compare several hundred maps (the size of the largest available data sets) in a reasonable time, which enables the user to test the robustness of the alignments by varying the event costs.

### 3.5. Implementation

Our algorithm is implemented in a program called MS_Align, which can be run through a web interface. For more details, we refer the reader to the section User’s guide of the website http://atgc.lirmm.fr/ms_align/. The distances matrix output by MS_Align can be directly used as input of standard phylogenetic reconstruction programs.

## 4. Biological validation on MSY1

In Section 3, we proposed an algorithm to align minisatellite maps under the evolutionary model detailed in Section 2. An optimal alignment of two maps represents a series of mutations needed to transform one map into the other. The alignment score can serve as a weighted measure of the evolutionary distance between these two maps. Thus, we wanted to know whether this measure is adequate to infer evolutionary relationships between the haplotypes represented by these maps. To this purpose, we tested our program on real biological data. We chose a large set of 609 MSY1 alleles of men originating from 76 different populations. The main reason for this choice resides in the availability of known evolutionary relationships for the Y chromosomes, and the knowledge of the Y-chromosomal haplogroup of each individual of the data set. Indeed, human Y chromosomal haplogroups have been defined from the analysis of more stable Y marker systems, mainly SNPs, and an evolutionary tree for these haplogroups have been proposed (Jobling and Tyler-Smith, 2000). Therefore, we used MS_Align to compute metric distances between pairs of alleles and infered phylogenetic trees from these distances using BIONJ (Gascuel, 1997). We could then investigate whether known phylogenetic relationships between Y chromosomes could be independently recovered from alignments between MSY1 MVR maps.

### 4.1. Data set

Mark Jobling provided us with the MSY1 maps of 690 individuals originating from 76 populations. Figure 1 displays examples of MSY1 maps. The MSY1 variants are 25 bases long and 7 different variants have been identified. Besides these variants, some maps display unidentified variants, which are termed *null repeat*. For example, this null repeat occurs in the alleles *m*47, *m*82, *m*121, *m*6, and *m*715 of Figure 1. We excluded the maps that contain more than 3 adjacent null repeats; this yielded a test set of 609 maps, with an average length of 70 repeats and less than 1 null repeat in average. On the Y chromosome, 27 haplogroups were defined using SNPs and other stable markers, and a most parsimonious tree was reconstructed for them (Jobling and Tyler-Smith, 2000). The MVR maps are identified by a code, their population of origin, and their haplogroup number.

Although a new nomenclature of Y chromosomal haplogroups has been published recently (The Y Chromosome Consortium, 2002), we use the nomenclature of (Jobling and Tyler-Smith, 2000) in the sequel. The new nomenclature has defined a larger number of haplogroups and inferred an evolutionary tree of their relationships using SNP data. However, the correspondence between the old and new nomenclatures is complex: for instance, the old haplogroup 2 is now split between clades B, G, and I of the new haplogroup tree. Without further information, it is not possible to determine to which of the new haplogroups an individual in our data set belongs, nor to compare our results to the haplogroup tree of new haplogroups. However, the data set based on the nomenclature of (Jobling and Tyler-Smith, 2000) is relevant for validation purposes.

The costs we used are *A* = *C* = 1, *D* = *I* = 40 and ℳ(*a*, *b*) = 10 ×*d*_H_ (*a*, *b*), where *d*_H_ (*a*, *b*) is the number of nucleotides that differ from variant *a* to variant *b*, *i.e.,* the Hamming distance between *a* and *b*. For MSY1, *d*_H_ (*a*, *b*) ≤ 3 ∀ *a*, *b* ∈ ∑. Since the introduction of a new letter can be done either by an insertion or by an amplification followed by a mutation, the value of *I* (and so *D*) is unimportant if *I* ≥ (*A* + ℳ(*a*, *b*)) ∀ *a*, *b* ∈ ∑, which is true for our application. The ratio ℳ(*a*, *b*)/*A* is important and we tried several ratios in our experiments. The best results are obtained for the values given above, in which the ratio corresponds approximatively to the value −log(mut.frequency/amp.frequency).

### 4.2. Haplogroup prediction with MSY1 maps

Using the matrix of pairwise distances between individuals and the *k*-nearest-neighbors method (Gordon, 1999), we predicted the correct haplogroup 80% of the time for *k* = 3 to 5 neighbors. Moreover, the percentage of time the correct haplogroup is within the 3 most represented haplogroups is about 93%. Always predicting the most probable class leads to a classification rate of 21.5%. In the same way, randomly predicting the class of the allele using the prior distribution of classes leads to a classification rate of 11.7%. This last rate is calculated as ∑*_i_*(|*C_i_*|/∑*_j_*|*C_j_*|)^2^, where |*C_i_*| denotes the cardinality of the class *C_i_*. The prediction based on the alignment distance between maps outperforms a random prediction, revealing the relation between the sequence of variants of an individual and its haplogroup.

### 4.3. An evolutionary tree of the Y-chromosome haplogroups derived from MSY1

From the matrix of distances between the individuals, we compute the matrix of average distances between the haplogroups. We then let BIONJ (Gascuel, 1997) reconstruct an evolutionary unrooted tree for the haplogroups with these distances as input. This tree is shown in Figure 5(*a*). We only include haplogroups for which at least 5 maps are available (1–4, 8–12, 15, 16, 18, 21, 22, 24, 26). A most parsimonious tree of the Y-chromosome haplogroups based on substitutional polymorphisms was proposed (Jobling and Tyler-Smith, 2000). A modified version of this tree, called SNP tree, appears on Figure 5(*b*). The modification consists in, first, restricting the tree to the haplogroups that also belong to our tree, and second, creating additional leaves for the haplogroups that label internal nodes in the original tree. This tree contains several polytomies, which prevent the direct comparison with our tree. Hence, we compute the number of leaves that should be removed from both trees in order to get maximal compatible subtrees (Page and Holmes, 1998), *i.e.,* the same subtrees except that a polytomy in the SNP tree can be replaced by a binary subtree in the other tree. For this, 4 removals are sufficient: haplogroups 4, 18, either 12 or 16, and either 21 or 8. This value of 4 leaves out of 16 shows how strongly the trees are related. The resulting compatible subtrees obtained with these removals can be seen as supplementary material.

In conclusion, the MSY1 tree agrees with the SNP tree for the most recent levels of evolution, which is consistent with the hypervariability of MSY1.

### 4.4. Internal evolution in two large populations

We constructed two trees for the Finnish and the Mongolian populations (Figures 6 and 7). A remarkable feature is that subsets of maps of the same haplogroup cluster together. Clearly, the structures of both trees reflect the relationships of the haplogroups in the MSY1 haplogroups tree, although the latter is based on average haplogroups distances. Hence, when the trees are estimated from the raw distances, they corroborate the partition in haplogroups and the relationships between these haplogroups.

### 4.5. Evolutionary relationships within the haplogroups

The Y chromosomes tend to be more closely related to one another inside a population than autosomal chromosomes. This and the difference in behavior between gender result in geographical specificity of the Y chromosomes. By computing evolutionary trees for the haplotypes within a haplogroup, we could check whether the alignment of MVR maps is able to recover a geographical clustering of the haplotypes.

For these experiments, we choose haplogroups that contain at least two different populations with at least two maps in each (otherwise it is impossible to detect a population separation). We focus on haplogroup 2 (European populations), 4 (Asian populations), and 16 (European and Asian populations). For haplogroup 2, which includes mainly European haplotypes, alleles of different populations are neighbors in the tree irrespective of their geographical origins (tree not shown). A reason for this is the fact that Europeans populations do not live isolated from each other and have largely exchanged their genetic material. The tree of haplogroup 4 (see Supplementary Material) perfectly separates 10 Japaneses from 3 Tibetans and 1 Mongolian. The Japanese haplotypes coalesce in a single clade. These populations live in geographically distinct area of Asia and their haplotypes cluster well in the tree.

The tree of haplogroup 16 (Figure 8) contains 57 MSY1 haplotypes, among which 3 populations are represented by several individuals: Mongolian (23), Finnish (10), Yakut and Siberian Yakut (13 + 5). In the tree, the Yakuts are monophyletic, all Finns but two form a monophyletic group, and the Mongolians agglomerate together (with one Japanese, two Finns, two Russians, and two Norwegians) and branch out between the Finnish and Yakut subtrees. Here, for haplogroup 16, whose number of haplotypes is much larger than haplogroup 4, we observe a high level of population specific coalescence. Apart from a few individuals in the Mongolian group, geographical separation appears clearly in this tree and agrees well with the geographical specificity of the Y.

## 5. Conclusion

Here, we presented a novel method for the alignment of minisatellite maps, which considers an extended evolutionary model with variable mutation costs. It improves in simplicity and in computational time upon previous solutions. Moreover, the program MS_Align can be used through a web-interface and is available upon request from the authors. We have applied our method on a large real data set from the human haploid hypervariable minisatellite MSY1. The alignment distance enables us to recover known phylogenetic relationships between Y-chromosomal haplogroups, showing the validity of the approach. In tentative experiments, we investigate the coalescence of alleles within haplogroups, and the outcome suggest the method could prove useful for micro-evolutionary studies. Our results highlight that the informativeness of minisatellites resides in their length and composition polymorphisms, which can both be exploited simultaneously.

MS_Align can be also used to analyze other types of tandem repeats.

To further validate our program for a wider range of minisatellite, we tested it with the variable GC-rich autosomal insuline minisatellite (INS). A study of the structural diversity of INS alleles could assign the alleles in three lineages called classes I, IIIA, and IIIB. Visual inspection and multidimensional scaling further divided class I into classes IC and ID (Stead and Jeffreys, 2000). In an experiment similar to those performed with MSY1 data, we compared the set of 181 INS alleles published in (Stead and Jeffreys, 2000) with MS_Align, and reconstructed a coalescence of these alleles from the resulting alignment distances (tree available as Supplementary Material). In this tree, the classes IC, ID, IIIA, and IIIB are all monophyletic, and the main split separates classes IC/ID from classes IIIA/IIIB. Again, by comparing the alleles of a variable GC-rich minisatellite, our alignment tool could infer automatically the distinct classes of alleles. This suggests that MS_Align could be well suited for deciphering the evolution of unstable, but non hypervariable, minisatellites.

Tandemly repeated protein sequences are also amenable to analysis. In an other work, our program was succesfully applied to decipher the evolution of a large family of proteins that contain a variable tandem repeat in the N-terminal parts of their sequences, the Pentatricopeptide Repeat family in *Arabidopsis thaliana* (Rivals et al. 2006).

A limitation of MS_Align for minisatellite analysis is the restriction on duplication and contraction to operate on a single variant, and not on a block of consecutive variants. In cases of block duplications, MS_Align overestimates the allele distance. In both MSY1 and INS, some alleles provide evidence for block duplications (*i.e.,* presence of a repeated block of several variants). However, this did not prevent the inference of correct allele relationships from the distances computed by MS_Align. Two reasons may explain this. First, both at MSY1 and INS loci the frequency of such events remains limited compared to single variant duplications and contractions (Stead and Jeffreys, 2000, Andreassen et al. 2002). Second, as a consequence of the preponderance of single variant duplications, duplicated or contracted blocks may often be themselves a stretch of a single variant with few or no mutations. Thus, the overestimation made by MS_Align tends to approximate well the real distance.

To authorize block duplications/contractions in minisatellite alignment makes the evolutionary model more general and even more realistic, but increases the complexity of the alignment procedure (as in (Sammeth et al. 2005)). A major challenge for future developments will be to generalize the evolutionary model (*e.g.*, taking into account inter-allelic exchanges) and to design a pairwise or a multiple alignment algorithm that remains efficient in practice.

## Supplementary Material

**Figure 1:**
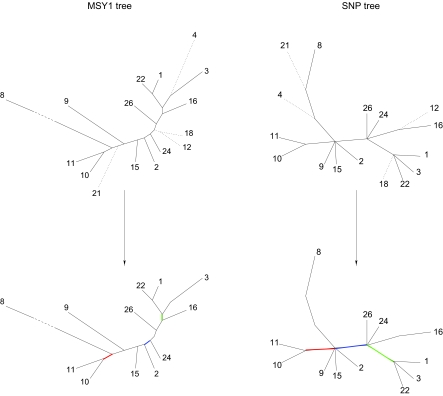
The Maximum Compatible Subtrees obtained by deleting leaves 4, 18, 12, 21 from the MSY1 haplogroups tree (left) and the SNP tree (right). When these deletions are applied to the two trees, the remaining subtrees contain the same leaves, but are not identical due to the polytomies in the SNP tree. This shows the similarity of the haplogroups tree of Figure 5 (in the article).

**Figure 2:**
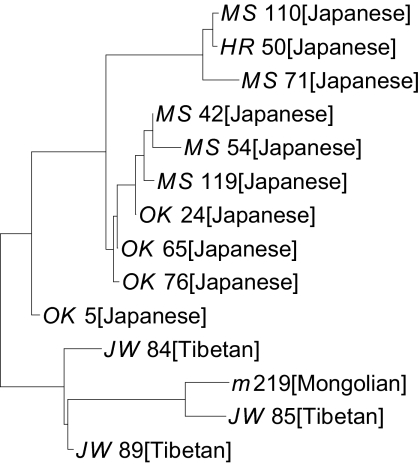
Phylogenetic tree of haplogroup 4. Each allele is given by its identifier and its population of origin. We compared the MSY1 alleles of this haplogroup pairwise with our algorithm, MS_Align(version 2), and used the resulting pairwise distance matrix to infer an evolutionary tree for the alleles using a Neighbor-Joining phylogenetic reconstruction program, BIONJ [Gascuel, 1997]. This tree perfectly separates 10 Japaneses from 3 Tibetans and 1 Mongolian. The Japanese haplotypes coalesce in a single clade.

**Figure 3:**
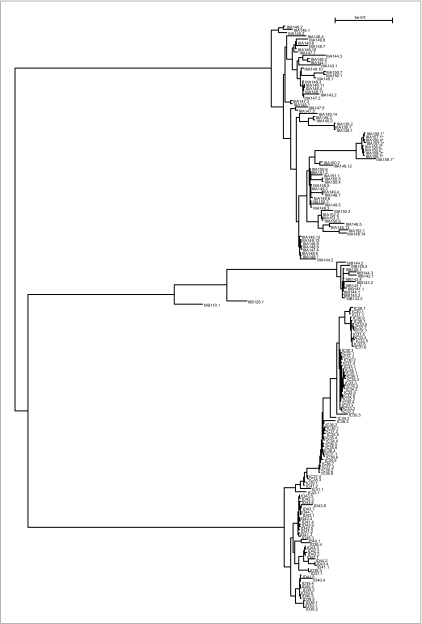
Coalescence tree of the 181 alleles of the insulin minisatellite published in [Stead and Jeffreys, 2000]. Alleles were compared with MS_Align to obtain a distance matrix which then serves as input for a distance based phylogenetic reconstruction program. The allele classes IC, ID, IIIA, and IIIB are monophyletic. In the tree, the top branch leads to the IIIA subtree, the second branch to the IIIB subtree, while the bottom branch is further split in two subtrees, one for class IC and one for class ID.

## Figures and Tables

**Figure 1:**
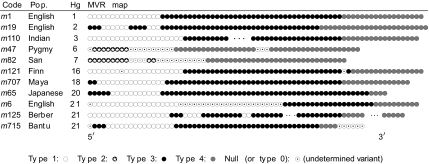
Examples of MSY1 maps of 11 individuals. The columns indicate the haplotype code, his population of origin, his haplogroup, and the MSY1 map (Jobling et al. 1998). For space reasons, the maps of the alleles *m*110 and *m*125 have been shortened by replacing a block of consecutive type 3 or type 4 variants by three dots.

**Figure 2:**
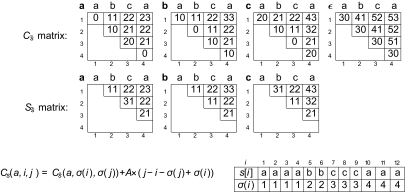
Example of the algorithm’s 1st phase with the RLE version of the map: *s* = *aaaabbcccaaa;* s̃ = *a*^4^*b*^2^*c*^3^*a^3^*; *A* = *C* = 1; *I* = *D* = 30; ℳ(*a*, *b*) = ℳ(*b*, *c*) = 10; ℳ(*a*, *c*) = 20. *Top:* computing the matrix 𝒞_s̃_. *Bottom*: formula to obtain the values of 𝒞*_s_* from *C_s̃_*.

**Figure 3:**
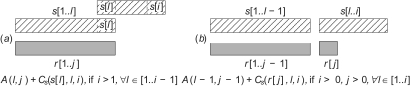
Second phase of calculation. Two possible segmental compressions: (*a*) compress *s*[*l..i*] in *s*[*l*] or (*b*) compress *s*[*l..i*] in *r* [*j*].

**Figure 4:**
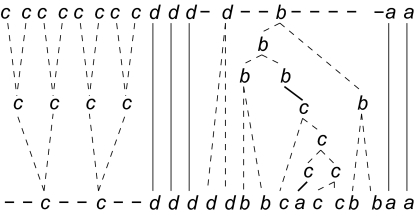
An alignment produced by MS_Align: the alignment between the maps *ccccccccddddbaa* and *ccdddddbbcaccbbaa* with the costs *A* = *C* = 1, *I* = *D* = 40, ℳ(*a*, *b*) = ℳ(*a*, *d*) = ℳ(*b*, *d*) = 20, ℳ(*a*, *c*) = ℳ(*b*, *c*) = ℳ(*c*, *d*) = 10. The cost of this alignment is 14 × *A* + ℳ(*b*, *c*) + ℳ(*c*, *a*) = 34. Plain lines represent matches, dashed lines represent amplifications and contractions, while bold lines represent mutations. One observes several segmental operations: first the compressions of the *c*’s at the beginning of the upper sequence, and the generation of the substring *bbcaccbb* in the lower sequence from the character *b* of the upper sequence. The latter shows a complex succession of elementary operations.

**Figure 5:**
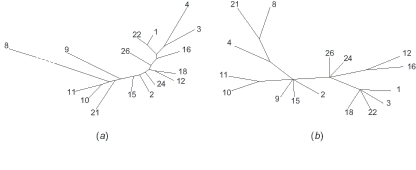
**(*a*)** The haplogroup tree infered from inter-haplogroup average distances between MSY1 alleles. We compared all MSY1 alleles pairwise with our algorithm and computed interhaplogroup average distances. We reconstructed an evolutionary tree from these distances using the distance based phylogenetic reconstruction program, BIONJ (Gascuel, 1997). **(*b*)** The haplogroup tree reconstructed from other stable markers (termed SNP tree) modified from (Jobling and Tyler-Smith, 2000). In both trees, the haplogroups are denoted by their number following the nomenclature of (Jobling and Tyler-Smith, 2000).

**Figure 6:**
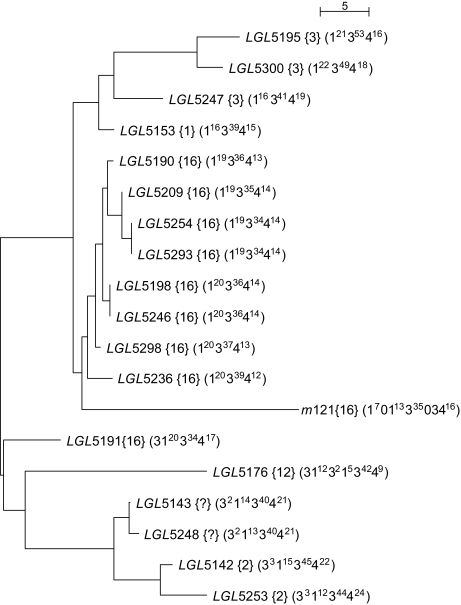
Phylogenetic tree of the Finnish population. Each haplotype is described by its code and its haplogroup (between braces). The MVR map of each haplotype is shown in its Run-Length encoded form between parentheses: a map is a sequence of stretches of identical variants. Each stretch is represented by the variant type number (as in Figure 1) with a power equal to the number of variants in the stretch; *e.g.,* 1^19^ meaning a stretch of 19 variant of type 1. For example, the map of the individual *m*121, displayed in Figure 1, is represented by 1^7^01^13^3^35^034^16^. Note that the haplogroup of haplotypes *LGL*5248 and *LGL*5143 is undetermined.

**Figure 7:**
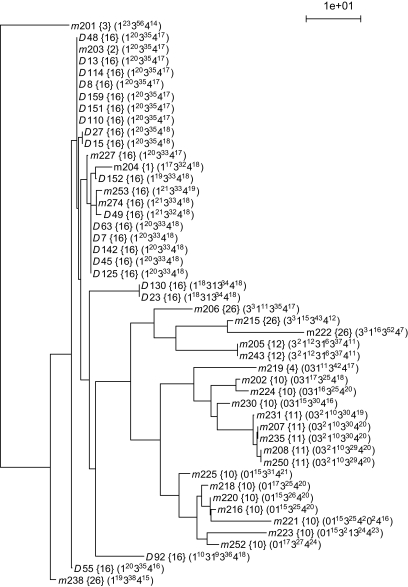
Phylogenetic tree of the Mongolian population. Each haplotype is described by its code and its haplogroup (between braces). The map of each haplotype is shown between parentheses (see Figure 6 for description).

**Figure 8:**
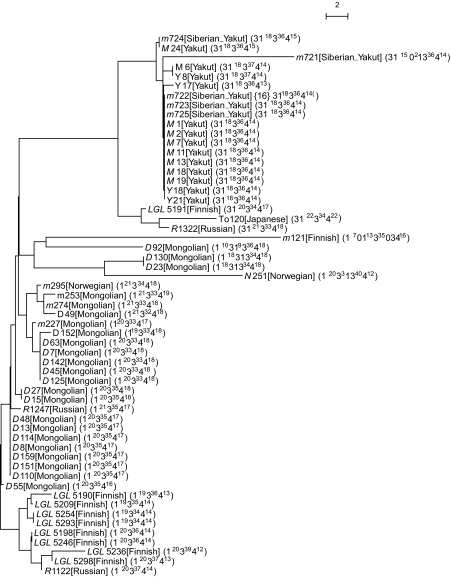
Phylogenetic tree of haplogroup 16. For each haplotype, we give its code, its population of origin between square brackets, and its MVR map between parentheses (see Figure 6 for description). The maps of this haplogroup were compared pairwise with MS_Align, and the resulting distance matrix was used to infer an haplotype evolutionary tree using a Neighbor-Joining method (Gascuel, 1997). Among the 57 maps of haplogroup 16, most belong to three main populations: Mongolian (23), Finnish (10), Yakut and Siberian Yakut (13 + 5). The Yakuts are monophyletic, all Finns but two form a monophyletic group, and the Mongolians agglomerate together (with one Japanese, two Finns, two Russians and two Norwegians) and branch out between the Finnish and Yakut subtrees.

## Footnotes

1 Independently of the costs *A* and ℳ(*a*, *b*) one can always find this kind of example by varying the size of the *a* blocks.
